# Human TRPM8 and TRPA1 pain channels, including a gene variant with increased sensitivity to agonists (TRPA1 R797T), exhibit differential regulation by SRC-tyrosine kinase inhibitor

**DOI:** 10.1042/BSR20140061

**Published:** 2014-08-06

**Authors:** Kevin Morgan, Laura R. Sadofsky, Christopher Crow, Alyn H. Morice

**Affiliations:** *Respiratory Medicine, Centre for Cardiovascular and Metabolic Research, Daisy Building, University of Hull and Hull York Medical School, Castle Hill Hospital, Cottingham HU16 5JQ, East Yorkshire, U.K.

**Keywords:** EC_50_, SNP, TRPA1, TRPM8, tyrosine kinases, AITC, allyl-isothiocyanate, A1TRPM8, transient receptor potential M8, AMTB, *N*-(3-Aminopropyl)-2-[(3-methylphenyl)methoxy]-*N*-(2-thienylmethyl)benzamide, HC 030031, 2-(1,3-dimethyl-2,6-dioxo-1,2,3,6-tetrahydro-7H-purin-7-yl)-*N*-(4-isopropylphenyl)acetamide, HEK, human embryonic kidney cells, ICL-1, intracellular loop-1, PP2, 4-amino-5-(4-chlorophenyl)-7-(t-butyl)pyrazolo[3,4-*d*]pyrimidine, PP3, 4-amino-7-phenylpyrazol[3,4-*d*]pyrimidine, RFU, relative fluorescence unit, SNP, single nucleotide polymorphism, TRPA1, transient receptor potential, WS 12, (1R,2S)-*N*-(4-methoxyphenyl)-5-methyl-2-(1-methylethyl)cyclohexanecarboxamide

## Abstract

TRPM8 (transient receptor potential M8) and TRPA1 (transient receptor potential A1) are cold-temperature-sensitive nociceptors expressed in sensory neurons but their behaviour in neuronal cells is poorly understood. Therefore DNA expression constructs containing human TRPM8 or TRPA1 cDNAs were transfected into HEK (human embryonic kidney cells)-293 or SH-SY5Y neuroblastoma cells and G418 resistant clones analysed for effects of agonists and antagonists on intracellular Ca^2+^ levels. Approximately 51% of HEK-293 and 12% of SH-SY5Y cell clones expressed the transfected TRP channel. TRPM8 and TRPA1 assays were inhibited by probenecid, indicating the need to avoid this agent in TRP channel studies. A double-residue mutation in ICL-1 (intracellular loop-1) of TRPM8 (SV762,763EL, mimicking serine phosphorylation) or one in the C-terminal tail region (FK1045,1046AG, a lysine knockout) retained sensitivity to agonists (WS 12, menthol) and antagonist {AMTB [*N*-(3-Aminopropyl)-2-[(3-methylphenyl)methoxy]-*N*-(2-thienylmethyl)benzamide]}. SNP (single nucleotide polymorphism) variants in TRPA1 ICL-1 (R797T, S804N) and TRPA1 fusion protein containing C-terminal (His)_10_ retained sensitivity to agonists (cinnamaldehyde, allyl-isothiocyanate, carvacrol, eugenol) and antagonists (HC-030031, A967079). One SNP variant, 797T, possessed increased sensitivity to agonists. TRPA1 became repressed in SH-SY5Y clones but was rapidly rescued by Src-family inhibitor PP2 [4-amino-5-(4-chlorophenyl)-7-(t-butyl)pyrazolo[3,4-*d*]pyrimidine]. Conversely, TRPM8 in SH-SY5Y cells was inhibited by PP2. Further studies utilizing SH-SY5Y may identify structural features of TRPA1 and TRPM8 involved in conferring differential post-translational regulation.

## Abstract 2

Transient receptor potential channels M8 and A1 sense the cellular environment and activate entry of Ca^2+^ into the cytoplasm. We identified a TRPA1 (transient receptor potential A1) variant with increased sensitivity and differential modulation of channels by a tyrosine kinase inhibitor in neuroblastoma cells.

## INTRODUCTION

Various human TRP (transient receptor potential) channels, encoded by a multi-gene family, modulate cell function by initiating transient elevations of intracellular Ca^2+^ concentration [1]. Although their fundamental biochemical properties are now well understood, aspects of TRP channel function in specialized cell types [2] and the potential importance of channelopathies remain incompletely characterized [3]. Consequently, roles for particular TRP channel mutants and their influence on therapeutic response requires elucidation [4,5].

Sensory neurons are a primary site for activation of TRP channel nociceptors by the external environment or endogenous factors. For example, TRPM8 (transient receptor potential M8) and TRPA1 (transient receptor potential A1) affect human physiology by initiating responses to volatile chemical agents or cold air via Ca^2+^-mediated activation of the peripheral nervous system, epithelia and underlying tissues [6–8]. They are modulated by specific biochemical species [9,10] or cell stress [11,12] and both TRP channels may be involved in pathological conditions such as asthma, chronic obstructive pulmonary disease and chronic cough [13–16]. Hence they are emerging targets for pharmacological intervention [17].

The basic characteristics of TRPM8 and TRPA1 channel biology were established, in part, by overexpression in cell line models, such as Chinese hamster ovary cells and human HEK (human embryonic kidney cells)-293 cells, coupled to measurements of transient elevations of intracellular Ca^2+^. Such studies enabled the identification of multiple agonists and antagonists [18–23] and identified the channel-modulatory effects of environmental conditions such as low external pH [24]. Site-directed or deletion mutagenesis and protein domain-swapping experiments, sometimes coupled with epitope- or GFP (green fluorescent protein)-tagging of recombinant channels, have revealed key amino acid residues and domains influencing TRPM8 and TRPA1 function (Table 1) [25–29]. Despite the accumulation of *in vivo* physiological data from animal models and the identification of interesting species-specific differences [30] (and in contrast to advanced molecular analyses of TRPV1 [31]), only few studies have examined the functional effects of recently catalogued genetic polymorphisms affecting the protein structure of human TRPM8 or TRPA1 [32,33]. Equally, the effects of experimentally designed structural modifications on the function of channel variants in different human cell types, including residues putatively involved in post-translational regulation [34–37], remain to be explored.

**Table 1:** Short list of functionally informative mutations in mammalianTRPM8 and TRPA1 (excluding N-terminal domain)

Location	TRPM8	TRPA1
S2 Y745	Menthol binding (mouse) [18]	
ICL-1	SV 762,763 EL retains function (this study)	
		R797T
		T alters EC_50_ (this study)
		S804N
		N, no effect on EC_50_ (this study)
S3		
ECL-2		
ICL-2		N855S (episodic pain) [43]
		S873E, E failed to express (this study)
S4	N799E/Y loss of icilin sensitivity [29]	
	D802A	
	G805A	
S5		
Pore domain	N934 essential glycosylation site [33]	
	C929, C940 required for activity	
S6		S943, I946 critical for gating [36]
C-ter domain	FK 1045,1046 AG retains function (this study)	
	1064 L–1104 K	1077E, 1080D, 1082D
	Coiled-coil tetramerization domain [32]	Regulation by Ca^2+^ [28]

Studies in neuronal-like cell models may be of great importance, for example, to understand the potential role of altered TRP channel regulation in the afferent neuronal hyper- or hyposensitivity characteristic of many disease states. Human neuronal cell models will also be required to evaluate data from the studies of TRP channels endogenously expressed in the peripheral nervous system of animals. In this respect, SH-SY5Y cells, a human neuroblastoma line capable of neurotransmitter biosynthesis [38] and *in vitro* neuronal dendrite extension, were recently stably transfected with TRPV1 [39,40], but studies with TRPM8 and TRPA1 have not been reported previously.

Therefore we compared stably transfected HEK-293 and SH-SY5Y cell clones expressing either normal or novel mutants of human TRPM8, and naturally occurring SNPs (single nucleotide polymorphisms) that generate sequence variants of TRPA1, alongside a C-terminally extended poly-His tagged TRPA1 fusion protein. We focused primarily on modifications affecting ICL-1 (intracellular loop-1) because this is a small domain likely to perturb channel function when structurally modified, but included modifications remote from ICL-1 for comparison. Pharmacological and functional properties of these channels were determined in both cell types.

## MATERIALS AND METHODS

### Reagents

The potent TRPM8 agonist WS 12 [(1R,2S)-*N*-(4-methoxyphenyl)-5-methyl-2-(1-methylethyl)cyclohexanecarbo-xamide], the TRPM8 antagonist AMTB [*N*-(3-Aminopropyl)-2-[(3-methylphenyl)methoxy]-*N*-(2-thienylmethyl)benzamide] hydrochloride and TRPA1 antagonists HC 030031 [2-(1,3-dimethyl-2,6-dioxo-1,2,3,6-tetrahydro-7H-purin-7-yl)-*N*-(4-isopropylphenyl)acetamide] and A967079 were from Tocris bioscience. Most other reagents, including menthol, ionomycin (A23187) Ca^2+^ ionophore, probenecid, sulfinpyrazone, bradykinin acetate and the TRPA1 activators [cinnamaldehyde, AITC (allyl-isothiocyanate), carvacrol, eugenol] were from Sigma-Aldrich. Reagent stock solutions were prepared and serially diluted in DMSO and stored at −20°C, unless stated otherwise. Restriction endonucleases were from Thermo Scientific.

### cDNA expression constructs

Human TRPM8 cDNA was cloned by RT–PCR from human prostate cancer cell line LNCaP (from the American Type Culture Collection) by ligation into pcDNA3.1neo and its DNA sequence validated (L.R. Sadofsky, unpublished work). Normal TRPM8 cDNA was mutated using synthetic oligonucleotides (from Eurofins, MWG, Operon) to amplify full-length plasmid DNA with Pfu DNA polymerase (Thermo Scientific). Amplified DNA was used to transform competent *Esherichia coli* (Agilent) and individual plasmid clones were screened by diagnostic restriction enzyme digestion-agarose gel electrophoresis. The TRPM8 SV 762,763 EL mutant was identified using a SacI digestion (GAGCTC) of the plasmid sequence generated by the PCR primers: sense 5′ATGGATTTCCATGAGCTCCCACA CCCC 3′ and its complementary sequence. The TRPM8 FK 1045,1046 AG mutant was identified using NaeI digestion (GCCGGC) of plasmid DNA generated using PCR primers: sense 5′ TCTTCTGTCTGCTGTGCCGGCAATGA AGA CAA TGAG 3′ and its complementary sequence.

Human TRPA1 cDNA in pcDNA3.1neo [41] was mutated to create SNP variants using quick change PCR with appropriate primer pairs: R797T forward 5′ CAACAGAAAACGAATTA TT and reverse 5′ AATAATTCGTTTTCTGTTG, S804N forward 5′ ATGGATATAAACA ATGTTC and reverse 5′ GAACATTGTTTATATCCAT. Likewise, the experimental mutant S873E, in ICL-2 (intracellular loop-2), was created using PCR primers: forward 5′ TTGTTGAGGGA GACAGTTG and reverse 5′ CAACTGTCTCCCTCAACAA. For C-terminal extension, TRPA1cDNA was modified by excision of the 3′ section of the coding region (BamHI–XbaI digestion) and replacement with a BamHI–XbaI digested PCR amplified section containing codons for ten histidine residues (His)_10_ prior to the translation stop codon using T4 DNA ligase (Promega). PCR primers were: sense 5′ TTTAC AGGATCCCTTCAGCTCTC CATT 3′ and antisense 5′ AGACTCGAGAAGCTTA GTGGTGATGATGGTGGTGAT GATGATGGTGTGTTTT TGCCTT 3′. Cloned recombinant plasmid DNA was identified using diagnostic NheI–HindIII restriction enzyme digestion-agarose gel electrophoresis.

### Cell culture

HEK-293 cells stably transfected with a pcDNA3.1neo (Invitrogen) constructs containing cDNA for human TRPM8 or TRPA1 were grown in DMEM 10% (v/v)FBS, penicillin and streptomycin under 0.5 mg/ml G418 (PAA Laboratories GmbH) selection [41]. HEK-293 cells were maintained on matrigel (BD Biosciences) coated plasticware. HEK-293 or SH-SY5Y cells (LGC) were transfected in 6-cm diameter dishes using Fugene 6 (Promega) following the suppliers instructions. Following selection with G418, distinct clones were picked using cloning cylinders and sequentially expanded in 12-well plates, and then T25 and T75 flasks prior to performing functional analyses and generation of frozen stocks.

### Intracellular Ca^2+^ measurements

When cells reached approximately 80% confluence they were harvested for assays measuring Ca^2+^ transients in response to TRP channel activation. Following a brief wash with PBS, cells were detached from each T75 flask by soaking with 2 ml Hepes-buffered saline EDTA (10 mM Hepes pH7.4, 155 mM NaCl, 1.7 mM EDTA) for several minutes and were harvested by addition of 10 ml PBS with gentle agitation and transfer to a 25 ml universal. A sample was taken to calculate cell yield using a hemocytometer and the cells were pelleted by centrifugation at 1500 rpm for 4 min. The pellet was resuspended in isotonic buffer (145 mM NaCl, 5 mM KCl, 1 mM MgCl_2_, 1 mM CaCl_2_, 10 mM Hepes pH7.4, 10 mM glucose, a variable content of probenecid (0, 0.13, 0.26, 0.52, 1.0, 2.0 or 2.5 mM) or 0.18 mM sulfinpyrazone, with optional 10 mg/ml BSA (all from Sigma-Aldrich) at a density of 5×10^6^/ml and mixed with 2.5 or 5.0 μl Fluo-3AM (Invitrogen) from a 2.5 μg/μl stock solution prepared in (DMSO, Sigma-Aldrich). Cells were incubated in a 25 ml universal in the dark at room temperature with gentle rotary mixing (50 rpm) for 30–45 min and then washed by addition of 18 ml PBS followed by centrifugation and resuspension in isotonic buffer without BSA at 5×10^6^ cells/ml.

Aliquots of cell suspension (usually 100 μl) were used for measurement of intracellular Ca^2+^ transients using a PTI (Photon Technology International) 814 photomultiplier detection system interfaced with a desktop computer (PTI Inc.). Cell suspension for assay was added to 1.9 ml isotonic assay buffer (no BSA) in a 10×10×45 mm polystyrene cuvette (Sarstedt Ltd) containing a small magnetic flea for continuous stirring (~50 rpm), to bring the final volume to 2 ml, and placed within the photomultipler light box. The cuvette was illuminated using light at a wavelength of 506 nm (slit width 8 nm) and monitored using fluorescence detection at 526 nm (slit width 8 nm) coupled to FeliX GX software (PTI Inc). Slit apertures were adjusted to enable maximum sensitivity while maintaining low noise. TRP channel agonist was administered by injection into the cuvette using a 1–20 μl pipette fitted with a narrow, extended-reach disposable tip. Antagonists were pre-added to cuvettes where appropriate. Real-time fluorescence intensity measurements were collected and data were exported to Microsoft Excel for analysis. Experimental treatments were performed in triplicate on three separate occasions to facilitate statistical analyses of reproducibility.

### Graphical and statistical analyses

Data from the measurement of Ca^2+^ transients (each treatment including a 2 μM A23187 Ca^2+^ ionophore control) were used to generate fluorescence intensity traces in Microsoft Excel. RFU (relative fluorescence unit) values for baseline and agonist-induced peak Ca^2+^ fluo-3-fluorescence were determined by the manual analysis of graphs. Agonist dose-dependent changes in peak RFU were expressed as ΔRFU relative to baseline and relative to maximum RFU elicited by A23187. This data were used to draw dose–response curves using nonlinear-regression curve fitting with GraphPad Prism software (GraphPad). This enabled estimation of excitatory concentration EC_50_ for agonists and inhibitory concentration IC_50_ values where pre-incubation with antagonist was employed. Comparisons between data generated in triplicate within individual experiments and between experiments performed on four separate occasions were analysed using Student's *t* test and one-way ANOVA with *post-hoc* analysis of group means using Tukey's multiple comparison test.

## RESULTS

Plasmid cDNA expression constructs for normal or mutant TRPM8, or TRPA1 variants and C-terminal fusion protein were prepared and transfected into HEK-293 and SH-SY5Y cell lines. Individual clones were isolated and expanded following selection for G418 resistance. SH-SY5Y cell growth was slower than HEK-293, with approximate cell doubling times of 48 and 24 h, respectively.

Cell clones were screened for expression of functional TRPM8 or TRPA1 channels using a fluo-3-based intracellular Ca^2+^ assay. Both cell lines activated fluo-3-AM efficiently in the absence of serum or BSA in isotonic buffer within 30–45 min at room temperature.

A total of 45 HEK-293 and 85 SH-SY5Y cell clones were screened and a proportion of them expressed the transfected TRP channel (Supplementary Tables S2 and S3 at http://www.bioscirep.org/bsr/034/bsr034e131add.htm). The success rate for isolation of SH-SY5Y clones expressing functional TRP channel constructs was lower than with HEK-293 cells. Attempts to isolate SH-SY5Y expressing the TRPM8 SV 762,763 EL mutant failed, despite screening multiple clones. None of the HEK-293 clones transfected with TRPA1 mutant 873E showed elevated intracellular Ca^2+^ in response to agonist treatment (20 clones were tested).

**Figure 1:**
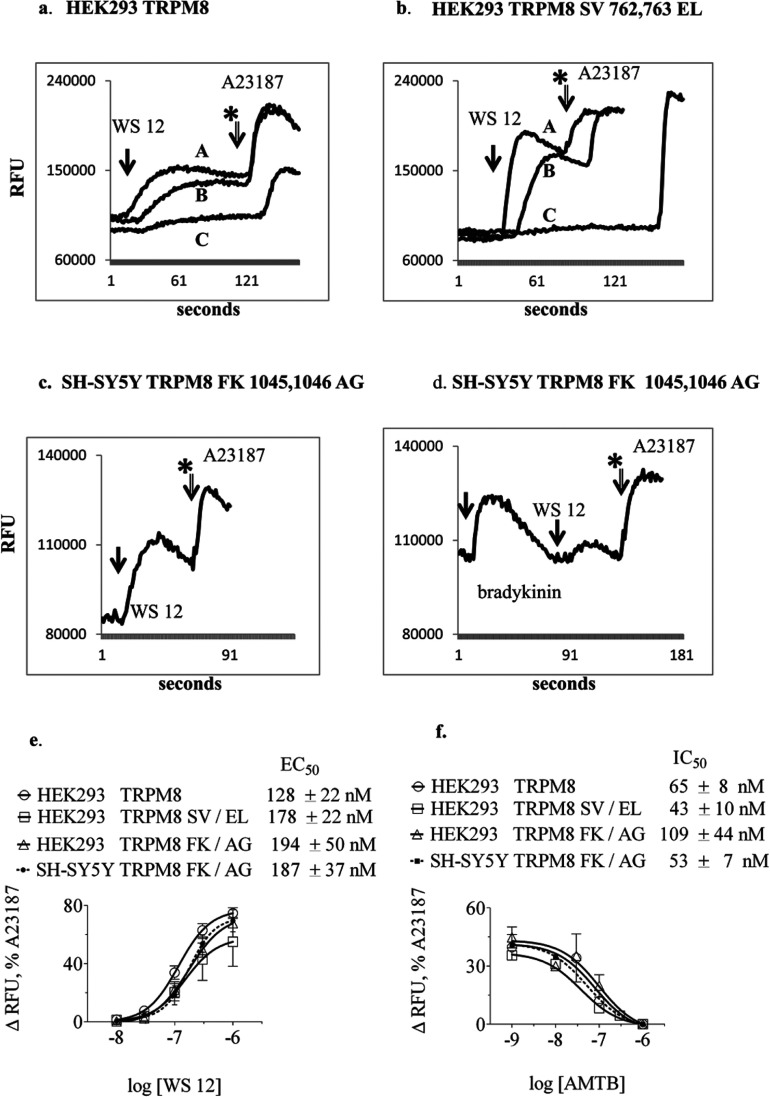
Representative fluorescence intensity traces from Fluo-3-loaded cells measuring real-time intracellular Ca^2+^ responses to TRPM8 agonist Relative fluorescence intensity units (RFU) are plotted versus time (s). (**a**) HEK-293 cells expressing human TRPM8 prepared in the presence of increasing concentrations of probenecid (A: 0.13 mM, B: 1.3 mM, C: 2.5 mM) treated with 40 nM WS 12 (injected at first arrow), then 2 μM A23187 (arrow with asterisk, indicated for first trace). Exposure to probenecid suppressed the response to WS 12. (**b**) Example Ca^2+^ transient traces showing dose-dependent WS 12 activation of TRPM8 SV 762,763 EL double residue mutant expressed in HEK-293 cells (A: 1 μM, B: 100 nM, C: 30 nM WS 12). (**c**) The Ca^2+^ transient response to 125 nM WS 12 in SH-SY5Y cells transfected with TRPM8 was similar in magnitude to the response to 2 nM bradykinin-mediated activation of endogenous bradykinin receptors, (**d**) (**e**) Dose–response curves derived from measurements of peak intracellular Ca^2+^ fluorescence in HEK-293 and SH-SY5Y cell clones expressing TRPM8 constructs. Estimation of EC_50_ for WS 12 (mean±S.E.M., *n*=3 independent experiments each performed in triplicate). Mean EC_50_ values for reference sequence and mutant TRPM8 were not statistically significant in either cell line. (**f**) Estimated IC_50_ values for TRPM8 antagonist AMTB determined using a constant dose of 150 nM WS 12, (mean±S.E.M., *n*=3). Mean IC_50_ values for wild-type and mutant TRPM8 were not statistically significant in either cell type.

High concentrations of probenecid (above 1 mM, and up to 2.5 mM), commonly used as an anion pump inhibitor to aid intracellular retention of fluo-3, inhibited TRPM8- and TRPA1-induced intracellular Ca^2+^ assays (Figure 1a and Supplementary Figure S1f at http://www.bioscirep.org/bsr/034/bsr034e131add.htm). Low probenecid concentration (0.13 mM) did not affect Ca^2+^ assay results relative to data derived using sulfinpyrazone. Following these observations, both probenecid and sulfinpyrazone were omitted from all subsequent assays since they were of no apparent benefit.

### Transfected HEK-293 and SH-SY5Y cell clones stably expressed functional human TRPM8 mutants SV 762,763 EL and FK 1045,1046 AG

Cell clones expressing mutant TRPM8 channels were compared with a stably transfected HEK-293 cell clone expressing normal human TRPM8. Each clone exhibited transient elevation in intracellular Ca^2+^ when treated with nanomolar concentrations of the potent TRPM8 synthetic agonist WS 12 (Figures 1b and 1c) or with micromolar menthol (results not shown).

Following a short equilibration period, baseline fluo-3-Ca^2+^ fluorescence during mixing of cells in suspension remained stable for up to (or more than) 10 min for each aliquot of cells, enabling multiple separate analyses from one batch of cells. The peak amplitude (change in RFUs, ΔRFU) of the fluo-3- Ca^2+^ fluorescence signal (526 nm) varied according to the concentration of WS 12 (Figure 1e) or menthol and depended on the concentration of extracellular Ca^2+^(results not shown). The time taken to reach peak amplitude lengthened as agonist concentration was lowered. Agonist-induced intracellular Ca^2+^ transients were specifically blocked by the potent TRPM8 antagonist AMTB, without effect on the A23187-induced changes (Figure 1b). Transient changes in intracellular Ca^2+^ elicited by WS 12 were similar to those elicited by bradykinin acting on endogenously expressed receptors (Figure 1d). Control HEK-293 cells transfected with empty pcDNA3.1neo vector did not exhibit intracellular Ca^2+^ transients following treatment with WS 12 (Supplementary Figure S1a), menthol or vehicle (DMSO; results not shown).

The baseline fluorescence signal intensity varied between experiments according to the number of cells loaded with fluo-3AM, the amount of fluo-3AM added to the cells and the duration of incubation with the dye prior to the experimental treatments. Therefore comparisons of peak RFU amplitude for each Ca^2+^ transient initiated by different treatments were made in close temporal proximity and on several different occasions to avoid erroneous interpretation of results, and data were standardized relative to the response to 2 μM A23187 for each sample. This enabled estimates of agonist EC_50_ values and antagonist (AMTB) IC_50_ values for TRPM8 mutants. Values for TRPM8 mutants expressed in either cell type were similar to normal TRPM8 channels (Figures 1e and 1f) and did not exhibit statistically significant differences. Inter-assay and intra-assay variability was monitored in triplicate and accounted for up to 25% variability in estimates of EC_50_ values and up to 40% variability in estimates of IC_50_ values. Values for peak ΔRFU as a percentage of the A23187-induced peak were similar for the different clones (closely spaced curves in Figures 1e and 1f).

### Transfected HEK-293 and SH-SY5Y cell clones expressed functional human TRPA1 SNP variants and TRPA1-(His)_10_ C-terminally extended protein

**Figure 2:**
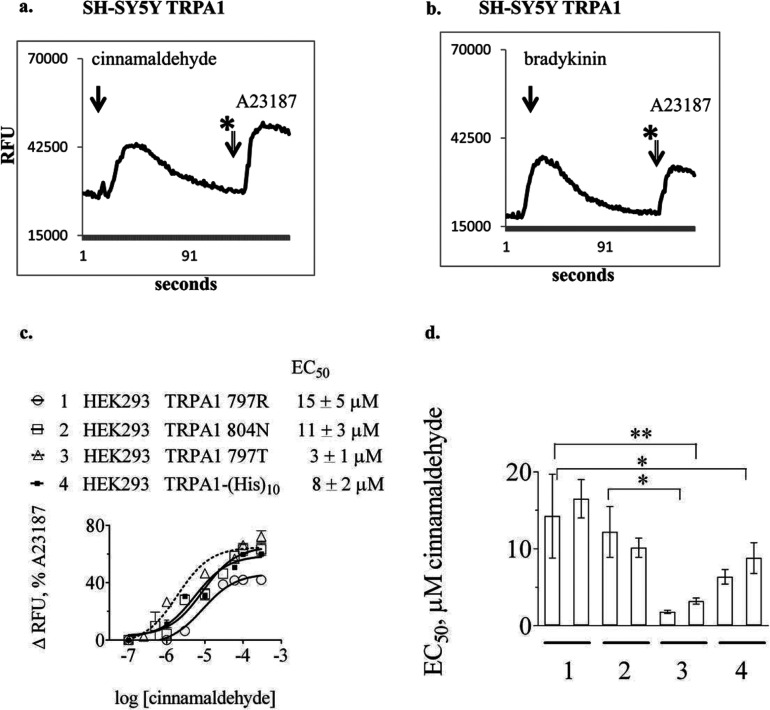
Representative fluorescence intensity trace from fluo-3 loaded transfected SH-SY5Y cells measuring real-time change in intracellular Ca^2+^ levels in response to TRPA1 agonist (**a**) (30 μM cinnamaldehyde) compared with the response to 2 nM bradykinin (**b**). (**c**) Dose–response curves for 1:TRPA1 797R, 2: 804N, 3: 797T and 4: TRPA1-(His)_10_ expressed in HEK-293 cells and treated with cinnamaldehyde. Mean EC_50_ (±standard deviation, *n*=4 separate experiments containing triplicate measurements on each occasion). EC_50_ values were 1: TRPA1 797R 15±5 μM, 2: TRPA1 804N 11±3 μM, 3: TRPA1 797T 3±1 μM and 4: TRPA1-(His)_10_ 8±2 μM. (**d**) Bar graph illustrating inter-experiment variation in estimation of EC_50_ for TRPA1 constructs 1–4. Data from duplicate experiments is shown (i.e. 2 bars per TRPA1 construct), in which measurements were performed in triplicate on each occasion. TRPA1 797T exhibited significantly lower EC_50_ compared with the other TRPA1 constructs,*indicates *P*<0.05, **P<0.01.

Individual cell clones expressing different TRPA1 proteins were isolated. Several clones were compared with each other using micromolar concentrations of agonist (cinnamaldehyde) and antagonists (HC 030031 or A967079) (Figures 2a, 2c and 2d and Supplementary Figures S1g–S1k). Effects on levels of intracellular Ca^2+^ were comparable with those elicited by activation of bradykinin receptors endogenously expressed in each cell line (Figure 2b).

Estimates of EC_50_ and IC_50_ values for TRPA1 SNP variants were made using HEK-293 cells (Figures 2c and 2d). Responses in different clones measured on different occasions were comparable (Figure 2d). The TRPA1 797T SNP variant exhibited a significantly lower EC_50_ in response to cinnamaldehyde (3±1 μM, *n*=4 repeats of triplicate measurements) compared with the reference sequence TRPA1 797R (Figure 2c) (15±5 μM) and compared with another SNP variant, TRPA1 804N (11±3 μM). Differences in EC_50_ response to cinnamaldehyde between the TRPA1 reference sequence and SNP 804N were not statistically significant, although the TRPA1-(His)_10_ protein appeared marginally more sensitive (EC_50_ 8±2 μM cinnamaldehyde). The increased sensitivity associated with TRPA1 797T was reproducible irrespective of which agonist was employed (cinnamaldehyde, AITC, carvacrol or eugenol, Supplementary Figure S1). Estimates of IC_50_ values for the potent antagonist A967079 were similar for each TRPA1 SNP variant investigated, with no statistically significantly differences being detected (Supplementary Figure S1k).

### TRPA1 and TRPM8 exhibited differential post-translational regulation in SH-SY5Y cells

**Figure 3:**
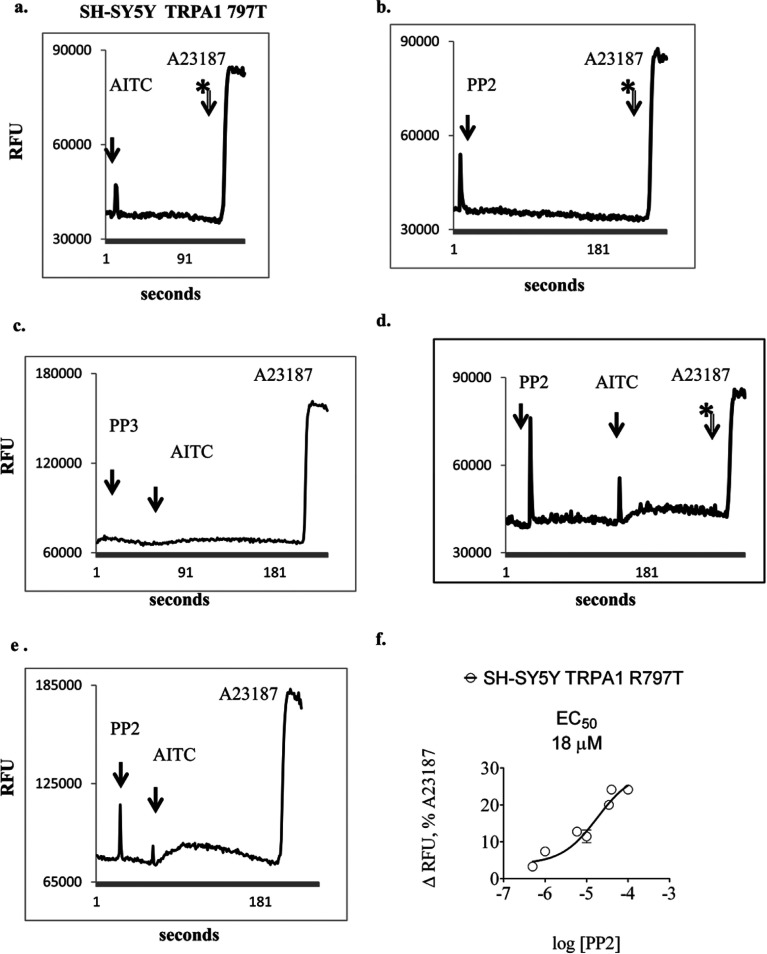
Representative traces for the real-time measurement of intracellular Ca^2+^ in transfected SH-SY5Y cells which exhibited loss of response to TRPA1 agonists (**a**) Cells were treated with agonist (6 μM AITC) followed by 2 μM A23187, (**b**) treated with Src family tyrosine kinase inhibitor PP2, (**c**) PP3, an inactive analogue of PP2 followed by AITC, or (**d**, **e**) PP2 followed by AITC. TRPA1 agonist induced intracellular Ca^2+^ elevation was poor in the absence of PP2 and increased following pre-treatment with PP2 in a dose-dependent fashion, with an EC_50_ of approximately 18 μM, (**f**) experiments were repeated on more than three separate occasions.

SH-SY5Y clones transfected with TRPA1 cDNA gradually lost detectable response to agonist following continuous *in vitro* culture for more than approximately 10–15 passages (Figures 3a and 3c). Response to agonist could be retrieved rapidly, in a dose-dependent fashion, by short pre-treatment with the Src family tyrosine kinase inhibitor PP2 [4-amino-5-(4-chlorophenyl)-7-(t-butyl)pyrazolo[3,4-*d*]pyrimidine] (Figures 3d–3f). Treatment with PP2 alone did not elicit elevation in intracellular Ca^2+^ over the same time course (Figure 3b). Treatment with PP3 (4-amino-7-phenylpyrazol[3,4-*d*]pyrimidine), an inactive analogue of PP2, did not facilitate recovery of responses to TRPA1 agonists (Figure 3c). PP2 elicited half-maximal rescue of response to 6 μM AITC when applied at approximately 18 μM (Figure 3f).

**Figure 4:**
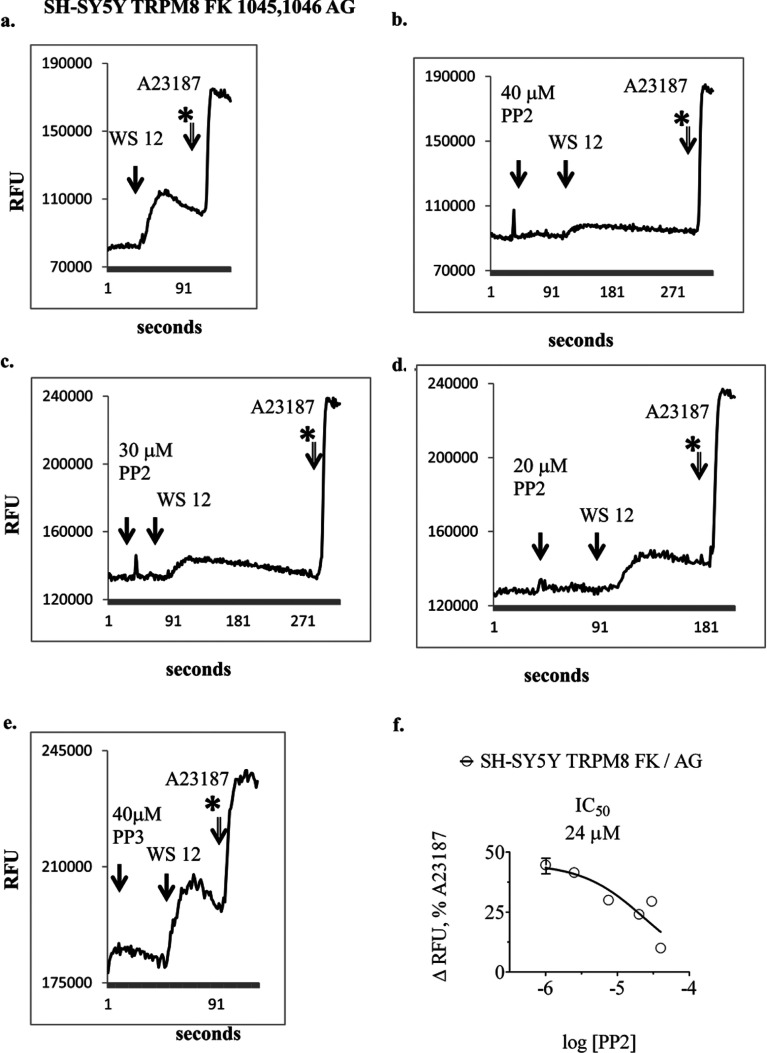
Representative traces for the real-time measurement of intracellular Ca^2+^ in SH-SY5Y cells transfected with TRPM8 FK 1045,1046 AG mutant (**a–e**) Pre-treatment with PP2 caused a dose-dependent inhibition of agonist -induced elevation in intracellular Ca^2+^ (150 nM WS 12). PP3 did not inhibit responses to agonist, (**e**) The IC_50_ for inhibition of TRPM8 response was approximately 24 μM PP2, (**f**) Dose–response experiments were repeated on more than three separate occasions.

SH-SY5Y clones transfected with TRPM8 did not exhibit gradual passage-dependent loss of response to agonist following continuous *in vitro* culture (Figure 4a). Furthermore, they responded to short pre-treatment with PP2 in an opposite fashion to TRPA1 clones. Here, PP2 dose-dependently inhibited responses to TRPM8 agonist (Figures 4b–4d). PP3 did not affect responses to TRPM8 agonist (Figure 4e). The half-maximal effect of PP2 occurred at approximately 24 μM (Figure 4f). In contrast to SH-SY5Y cells, treatment of HEK-293 cells with PP2 caused large elevations of intracellular Ca^2+^ levels that precluded analyses of effects on TRPA1 and TRPM8 responses (results not shown).

## DISCUSSION

In this study, we used site-directed mutagenesis, recombinant DNA engineering and measurements of agonist-induced Ca^2+^ influx to characterize TRPM8 and TRPA1 channel function in neuroblastoma cell culture. This is the first study to report expression of modified human TRPM8 or TRPA1 proteins in human SH-SY5Y neuroblastoma cells and we present several significant findings. The results suggest that SH-SY5Y cell studies may be a useful tool to explore neuronal cell-type-specific features of TRP channel behaviour through application of Ca^2+^ signalling assays and analyses of molecular interactions using epitope-tagged proteins.

We found that probenecid at millimolar concentrations inhibited assays for both TRPM8 and TRPA1, confirming and extending previous data [42]. This observation is of technical importance since certain *in vitro* pharmacological studies include probenecid in assay buffers. We recommend avoiding the use of probenecid in studies of TRPM8 and TRPA1.

Specific TRPM8 and TRPA1 experimental mutants were generated to address particular hypothetical questions. We focussed mainly on ICL-1 because it is one of the smallest structural domains in either channel. Mutations here might be expected to have important effects on channel tertiary structure, impinging on channel function. Although SNPs affecting ICL-1 in human TRPA1 have been catalogued, they have not been studied previously.

TRPM8 (SV 762,763 EL) is an engineered mutant, mimetic of hypothetical phosphorylation (S 762 E) at ICL-1 between transmembrane domains S2 and S3. The phosphorylation of channels, usually at multiple serine/threonine or tyrosine residues, is a common post-translational regulatory mechanism. Likewise, intracellular lysine residues can be used as sites of regulatory modification, such as ubiquitination. TRPM8 FK 1045,1046 AG, is essentially a lysine knockout (K 1046 G) in the C-terminal tail region (remote from ICL-1) and is the first of its kind studied in TRPM8.

TRPA1 R797T and S804N are naturally occurring SNP variants affecting residues in ICL-1. These were chosen because the former is a non-conservative substitution and the latter a semi-conservative substitution and they are suitable for comparison with each other. In our hands, an experimental mutant in TRPA1 ICL-2, S873E, failed to yield responsive clones. Further investigation are required to determine whether this mutant can be expressed.

Interestingly, both TRPM8 mutants retained channel gating in response to agonist (WS 12 or menthol) and sensitivity to antagonist (AMTB), with little impact on Ca^2+^ influx and no change on estimated EC_50_ or IC_50_ values (Figure 1). They provide a useful comparator with other TRPM8 mutants and TRPA1 SNPs.

In contrast to the absence of effect of mutation in ICL-1 of TRPM8, the TRPA1 ICL-1 SNP variant 797T exhibited greater sensitivity to agonists than the more common TRPA1 allele 797R (Figure 2) and compared with the TRPA1 SNP affecting position 804, also in ICL-1. This is a significant discovery, reminiscent of the putative sensitizing effect associated with an SNP affecting ICL-2, N855S [33]. Further studies are required to assess whether genetic variants contribute to physiological differences in TRPA1 sensitivity in humans.

Of equal importance, we discovered evidence for differential post-translational regulation of TRPA1 and TRPM8 in SH-SY5Y cells. This operated through a pathway involving tyrosine kinase activity (Figures 3 and 4), sensitive to the inhibitor PP2. The EC_50_ and IC_50_ values for the effects of PP2 on TRPA1 and TRPM8 were of a similar magnitude (about 20 μM). The inactive analogue PP3 did not affect TRP channel responses. Similar experiments could not be performed using HEK-293 clones because PP2 elicited large elevations in intracellular Ca^2+^ in these cells. An inhibitory effect of 10 μM PP2 on TRPV1 agonist-induced trans-membrane current in HEK-293 cells has been reported previously [43].

The TRPA1-(His)_10_ tag did not appreciably alter the expected EC_50_ of responses to cinnamaldehyde. Perhaps the use of epitope tags as tools to probe molecular interactions may be useful to examine whether the TRP channel mutants reported in this study have any currently unrecognized impact on channel behaviour. For example, chronically activated TRP channels are silenced, in part, by a combination of multiple phosphorylations and internalization by endocytosis (possibly modulated by ubiquitination), followed by recycling or proteolytic degradation. The molecular interactions mediating the equilibrium between these processes for each TRP channel subtype are generally poorly understood [36] and may be relevant to the long-term response of cells to treatment with antagonists.

In summary, our data indicate that rare naturally occurring polymorphisms can affect the sensitivity of TRPA1 and that SH-SY5Y cells may be useful for the study of post-translational regulation of TRPA1 and TRPM8 mutants in neuroblastoma cells operating via mechanisms potentially different to those present in HEK-293 cells.

## Online data

Supplementary data
