# Supplementary material for: Human TRPM8 and TRPA1 pain channels, including a gene variant with increased sensitivity to agonists (TRPA1 R797T), exhibit differential regulation by SRC-tyrosine kinase inhibitor
Source: Biosci Rep. 2014 Aug 6;34(4):e00131. doi: 10.1042/BSR20140061 (PMC4122973; doi:10.1042/BSR20140061)
Supplement: Supplementary data [file bsr034e131add.pdf]

Biosci. Rep. (2014) / **34** / art:e00131 / doi 10.1042/BSR20140061

OPEN ACCESS

Original Paper

Bioscience Reports

www.bioscirep.org

Bioscience Reports

SUPPLEMENTARY DATA

Human TRPM8 and TRPA1 pain channels, including a gene variant with increased sensitivity to agonists (TRPA1 R797T), exhibit differential regulation by SRC-tyrosine kinase inhibitor

Kevin MORGAN*[1], Laura R. SADOFSKY*, Christopher CROW* and Alyn H. MORICE*

*Respiratory Medicine, Centre for Cardiovascular and Metabolic Research, Daisy Building, University of Hull and Hull York Medical School, Castle Hill Hospital, Cottingham HU16 5JQ, East Yorkshire, U.K.

Table S1 TRPM8-transfected cell clones examined in this study

Cell line and expression construct	Incidence of clones responsive to agonist
HEK-293 cells	
pcDNA3.1 TRPM8 N/A	[41]
pcDNA3.1 TRPM8 SV 762,763 EL	3/6
pcDNA3.1 TRPM8 FK 1045,1046	AG 2/6
Success rate	5/12 = 42%
SH-SY5Y	
pcDNA3.1 TRPM8	1/16
pcDNA3.1 TRPM8 FK 1045,1046 AG	2/6
pcDNA3.1 TRPM8 SV 762,763 EL	0/3
Success rate	3/25 = 12%

Table S2 TRPA1-transfected cell clones examined or isolated in this study

Cell line and expression construct	Incidence of clones responsive to agonist
HEK-293 cells	
pcDNA3.1 TRPA1 797R N/A	[41]
pcDNA3.1 TRPA1-$(His)_{10}$	7/11
pcDNA3.1 TRPA1 797T	7/12
pcDNA3.1 TRPA1 804N	4/10
Success rate	18/33 = 55%
SH-SY5Y	
pcDNA3.1 TRPA1-$(His)_{10}$	2/6
pcDNA3.1 TRPA1 797T	1/30
pcDNA3.1 TRPA1 804N	4/24
Success rate	7/60 = 12%

[1] To whom any correspondence should be addressed (email K.Morgan@hull.ac.uk).

© 2014 The Author(s) This is an Open Access article distributed under the terms of the Creative Commons Attribution Licence (CC-BY) (http://creativecommons.org/licenses/by/3.0/) which permits unrestricted use, distribution and reproduction in any medium, provided the original work is properly cited.

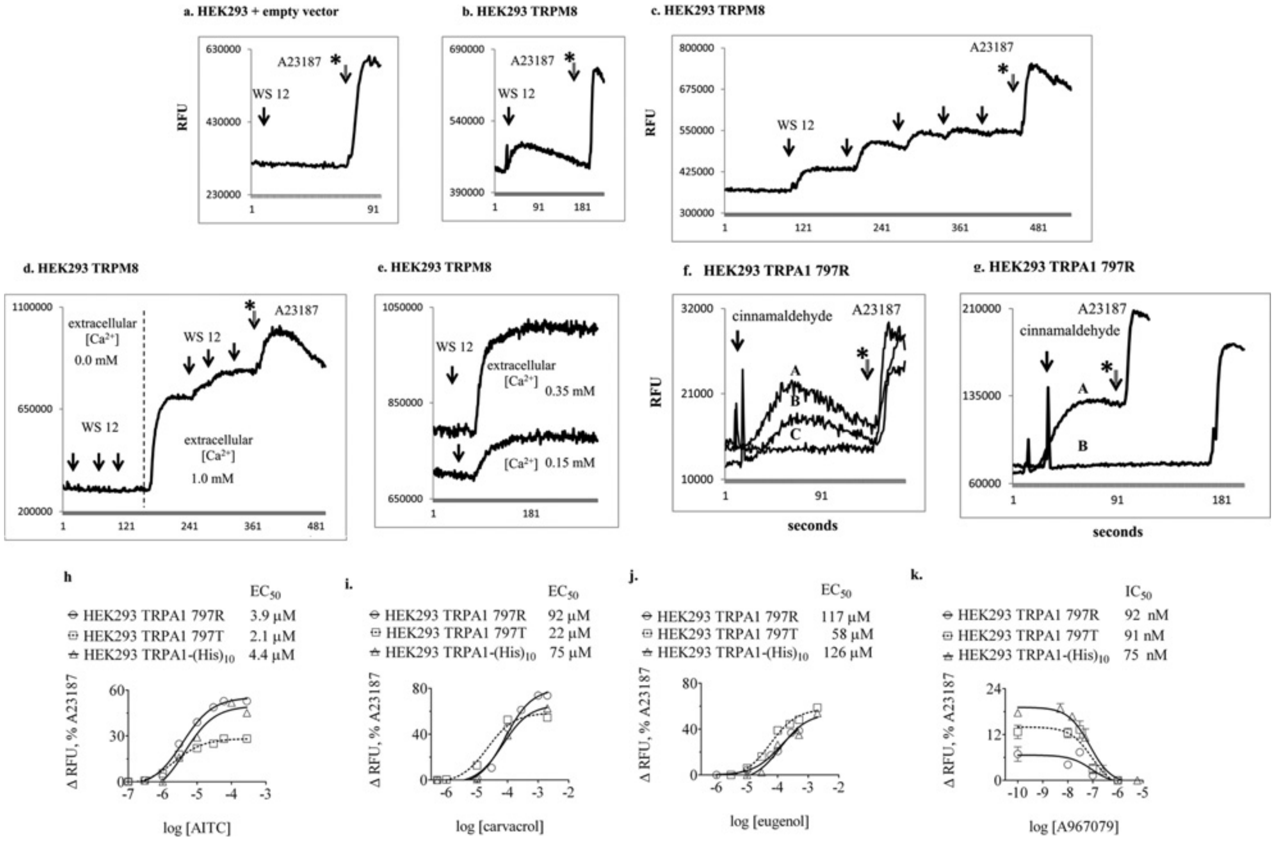

Figure S1 **Intracellular Ca^{2+} responses elicited via TRPM8 and TRPA1 and further estimates of EC_{50} and IC_{50} values**
(**a**) Absence of response to 40 nM WS 12 in HEK-293 cells transfected with empty vector DNA (pcDNA3.1 containing no cDNA insert). There is no alteration in baseline fluorescence relative to the peak elicited by A23187. (**b**) A response elicited by 40 nM WS 12 in HEK-293 cells transfected with TRPM8 expression construct for comparison with (**a**). (**c**) Sequential injection of five 40 nM doses of WS 12 elicited a cumulative fluorescence signal in HEK-293 cells transfected with TRPM8 expression construct, with evidence of a maximized effect following the fifth dose. (**d**) Injection of three 40 nM doses of WS 12 does not elicit a response from HEK-293 cells transfected with the TRPM8 expression construct when the cells were prepared in Ca^{2+}-free buffer. Subsequent addition of Ca^{2+} to 1 mM (dashed line) resulted in increased fluorescence that could be increased by further doses of 40 nM WS12. (**e**) The concentration of extracellular Ca^{2+} in the assay buffer affected the magnitude of fluorescence signal following addition of 40 nM WS 12 to HEK-293 cells transfected with the TRPM8 expression construct. (**f**) HEK-293 TRPA1 cell responses inhibited by probenecid. HEK-293 cells expressing human TRPA1 prepared in the presence of increasing concentrations of probenecid (A, 0.13 mM; B, 1.3 mM; C, 2.5 mM) treated with 60 μM cinnamaldehyde (injected at first arrow), and then 2 μM A23187 (arrow with asterisk). Exposure to probenecid suppressed the response to cinnamaldehyde. (**g**) HEK-293 TRPA1 cell responses inhibited by TRPA1 antagonist. Cells treated with 60 μM cinnamaldehyde, A, compared with cells pre-treated with 1 μM A967079 prior to 60 μM cinnamaldehyde, B. (**h–j**) Estimates of EC_{50} values for TRPA1 mutant responses to different agonists. (**k**) Estimates of IC_{50} values for TRPA1 mutants pre-treated with A967079 prior to 2 μM AITC.

Received 15 April 2014/2 May 2014; accepted 16 May 2014

Published as Immediate Publication 30 June 2014, doi 10.1042/BSR20140061

© 2014 The Author(s) This is an Open Access article distributed under the terms of the Creative Commons Attribution Licence (CC-BY) (http://creativecommons.org/licenses/by/3.0/) which permits unrestricted use, distribution and reproduction in any medium, provided the original work is properly cited.
